# Higher urine 1-hydroxy pyrene glucuronide (1-OHPG) is associated with tobacco smoke exposure and drinking maté in healthy subjects from Rio Grande do Sul, Brazil

**DOI:** 10.1186/1471-2407-6-139

**Published:** 2006-05-26

**Authors:** Renato B Fagundes, Christian C Abnet, Paul T Strickland, Farin Kamangar, Mark J Roth, Philip R Taylor, Sanford M Dawsey

**Affiliations:** 1 Universidade Federal de Santa Maria, Departamento de Clínica Médica, Centro de CIências da Saúde, Santa Maria, RS, Brazil; 2 Universidade Federal do Rio Grande do Sul, Gastroenterology Post Graduate Course. Porto Alegre, RS, Brazil; 3 Nutritional Epidemiology Branch, Division of Cancer Epidemiology & Genetics, National Cancer Institute, Bethesda, MD, USA; 4 Department of Environmental Health Sciences, The Johns Hopkins University Bloomberg School of Public Health, Baltimore, MD, USA; 5 Genetic Epidemiology Branch, Division of Cancer Epidemiology & Genetics, National Cancer Institute, Bethesda, MD, USA

## Abstract

**Background:**

The highest rates of esophageal squamous cell carcinoma (ESCC) in Brazil occur in Rio Grande do Sul, the most southern state, which has incidence rates of 20.4/100,000/year for men and 6.5/100,000/year for women. Exposure to carcinogenic polycyclic aromatic hydrocarbons (PAHs) through tobacco smoke and other sources may increase the risk of ESCC. The aims of the current study were to investigate the degree and sources of PAH exposure of the inhabitants of this region of southern Brazil.

**Methods:**

Two hundred healthy adults (half smokers, half non smokers, half male and half female) were recruited, given a standardized questionnaire, and asked to provide a urine sample for measurement of 1-hydroxypyrene glucuronide (1-OHPG), a PAH metabolite). Urine 1-OHPG concentrations were measured using immunoaffinity chromatography and synchronous fluorescence spectroscopy and urine cotinine was measured using a dipstick test. We examined factors associated with 1-OHPG concentration using Wilcoxon tests and multiple linear regression.

**Results:**

Urine 1-hydroxypyrene glucuronide (1-OHPG) was successfully measured on 199 subjects. The median (interquartile range) of urine 1-OHPG in the 199 participants was 2.09 pmol/mL (0.51, 5.84). Tobacco smoke exposure and maté drinking were statistically significantly associated with higher urine 1-OHPG concentrations in the multivariate linear regression model.

**Conclusion:**

Tobacco smoke and maté both contribute to high levels of benzo[a]pyrene exposure in the people of southern Brazil. This high PAH exposure may contribute to the high rates of ESCC observed in this population. The increased urine 1-OHPG concentrations associated with maté suggest that contaminants, not just thermal injury, may help explain the increased risk of ESCC previously reported for maté consumption.

## Background

Esophageal cancer is a common and usually fatal cancer that is characterized by great variation in rates among different populations. In South America, there is a geographic area of high esophageal squamous cell carcinoma (ESCC) incidence that encompasses southern Brazil, northeastern Argentina, Uruguay, and Paraguay, with age-standardized incident rates of approximately 20/100,000/year [1]. Inhabitants of this area share a similar environment and similar habits and culture. Two habits they have in common that may contribute to the high ESCC rates are the high consumption of grilled red meat called *churrasco *and a daily consumption of large volumes of a beverage known as *maté*. *Churrasco *is barbequed meat grilled directly over a wood fire and is a potential source of polycyclic aromatic hydrocarbons (PAH), heterocyclic amines, and other contaminants which may be associated with cancer in humans [2,3]. Mate is an infusion of the herb *Ilex paraguayensis *that is prepared in a gourd and is often drunk very hot through a metal straw, which delivers the liquid directly to the oropharynx and esophagus. Most epidemiologic studies that examined maté drinking have found significant associations with ESCC [4-10], but one reported no association [11].

In low-risk areas, most ESCC is attributable to alcohol, tobacco, and poor diet [12], but the etiologic agents in high-risk populations remain unclear. PAHs, such as benzo[a]pyrene, and nitrosamines from tobacco smoke and from other sources may act as esophageal carcinogens [2,13-17]. Animal studies have demonstrated a dose-response relationship between benzo[a]pyrene food levels and the incidence of esophageal cancer in mice [18].

Studies in the very high risk population of Linxian, China, where consumption of tobacco and alcohol is low, suggest that the inhabitants there may be exposed to high-levels of carcinogenic PAHs from the coal and wood used for cooking and heating in unvented stoves [19-21]. This hypothesis is also supported by the finding of high levels of benzo[a]pyrene in uncooked food samples [15], histological changes suggestive of PAH exposure in esophagectomy specimens [14], and high 1-hydroxypyrene glucuronide (1-OHPG) concentrations in urine samples from the inhabitants of this region [16]. 1-OHPG is a stable PAH metabolite that is excreted in the urine and is an index biomarker that reflects recent exposure to mixed PAHs [22-27]. In northeastern Iran, another area with very high rates of ESCC and little consumption of tobacco and alcohol, the population also has high urine 1-OHPG concentration, consistent with exposure to very high levels of PAHs [28].

To determine the degree and source of PAH exposure in inhabitants of southern Brazil we collected questionnaire data and determined urine concentrations of 1-OHPG from inhabitants of Rio Grande do Sul, the most southern state in Brazil.

## Methods

### Subjects

Participants were volunteers from Santa Maria, a city in the central region of Rio Grande do Sul. People attending the outpatient unit of the University Hospital with minor injuries, minor skin diseases, to donate blood, women visiting the gynecologist for annual screening, patients with dyspepsia after a normal upper gastrointestinal endoscopy, and patient's companions were considered eligible. After a brief description of the study purpose and requirements they were invited to participate. Greater than 90% of the invited individuals chose to participate. Subjects were recruited so that among the 200 healthy people half were male and half were female and within each of these groups half were current smokers and half were non-smokers. Informed consent was obtained from each participant. The study was approved by the Ethical Committee on Research of the Health Sciences Center of the University of Santa Maria, RS, Brazil, and the analysis of anonymized data and samples was exempted from review by the Institutional Review Board of the National Cancer Institute, Bethesda, MD.

### Questionnaire

All subjects were interviewed face-to-face using a pre-tested, standardized questionnaire, administered by specially trained interviewers. The questionnaire included: basic demographic variables and residence characteristics; habits of tobacco smoking (age started, age stopped, typical number of cigarettes per day, type of tobacco, and passive smoke exposure); history of alcohol drinking (type of alcoholic beverage, amount of each beverage consumed, duration of consumption); history of maté drinking (amount usually consumed/day); frequency of and fuel used to make barbeque and other cooking; preferred doneness and amount of barbeque typically eaten; and home heating fuel system and its smokiness.

### Urine measurements

Each participant was recruited in the morning asked to provide a 10 ml urine sample. The urine samples were collected in a sterile container, frozen at -80°C, and shipped on dry ice to the National Cancer Institute. Urine samples were assayed in the laboratory of Dr. Strickland at the Johns Hopkins University. Urine 1-OHPG concentrations were measured using immunoaffinity chromatography and synchronous fluorescence spectroscopy as previously described [16,25]. NicAlert Strips (Jant Pharmaceutical Corp., Encino, CA) were used to measure urine cotinine equivalents as directed by the manufacturer. This test produces categorical results ranging from zero (<1–10 ng/mL cotinine equivalents) to six (>2000 ng/mL). Because only a small number of subjects had urine cotinine results in each of categories two, three, and four, we collapsed these three groups into a single category.

### Statistical analysis

Urine 1-OHPG concentrations were examined graphically and found to be skewed with a mode at the limit of detection which included 37/199 (19%) subjects. Log_10 _transformation produced a normal curve outside the mode. Urine 1-OHPG was also represented as quintiles for some analyses. Univariate associations with 1-OHPG were examined by forming exposure data into quantiles and comparing them with the Wilcoxon rank sum test or the Kruskal-Wallis test. Age and maté were divided into empirical quartiles. Multivariate associations were examined using log-transformed urine concentrations in linear regression models. The final model was built by adding all variables, with selected members of a class (e.g. only one of the variables associated with barbeque preparation), and deleting those that were not significant, based on the F-test, and whose removal did not change the estimates for the remaining variables. Interactions between sex or tobacco smoking and other variables were explored. When tested independently (data not shown) we found a significant interaction between barbeque preparation and smoke exposure, so it was retained in the final model. A borderline significant interaction between smoke exposure and maté was also retained. The p-values for the interactions were inflated slightly in the final model. To better show the effects of these interaction we plotted the data for the four groups. Data for plotting was jittered to improve clarity. All analyses were carried out using SAS version 9 (SAS Institute, Cary, NC). All p-values come from two-sided tests.

## Results

We recruited a total of 200 subjects evenly divided by sex and current smoking status and each completed a questionnaire and provided a urine sample. We successfully measured urine 1-OHPG and cotinine on 199 of these subjects, so these subjects appear in the remaining analyses. The median (interquartile range) of urine 1-OHPG in the 199 participants was 2.09 pmol/mL (0.51, 5.84). Table 1 presents the distributions and univariate associations between personal characteristics and habits and the concentration of 1-OHPG. We found associations between urine 1-OHPG concentrations and age, tobacco use, urine cotinine, smoke exposure maté consumption, and drinking *cachaça *(distilled sugar cane liquor). Smoke exposure was defined as all subjects reporting current tobacco smoking or a cotinine value greater than category 1 (>30 ng/ml). Maté consumption showed a step-wise increase between volume consumed and urine 1-OHPG concentration

We used tabular analysis to look for associations between these factors and tobacco smoking, the most likely contributor to urine 1-OHPG. We found associations between tobacco smoking and each of the factors. By increasing order of age group, we found a prevalence of smoking of 52%, 63%, 58%, and 17%, respectively, (3 df chi-square < 0.0001). Fifty-three percent of maté consumers, but only 29% of non-consumers reported current smoking (chi-square *P *= 0.0031). We expected and found that most subjects with urine cotinine values of 5 or 6 reported current use of tobacco. Fifty-three percent of non-smokers had cotinine values greater than category zero, but only 1 had a value of 5 or 6. Significantly more *cachaça *drinkers (71%) reported current tobacco smoking than did non-drinkers (44%) (chi-square *P *= 0.0042).

Table 2 presents the results from a multivariate linear regression model for the association between the examined factors and urine 1-OHPG. The final model had a total r^2 ^of 0.21. Our multivariate model showed significant associations between urine 1-OHPG and age, smoke exposure, maté drinking, but the univariate association with *cachaça *appears to have resulted from confounding by tobacco smoke exposure. We found interactions between smoke exposure and both maté drinking and barbeque preparation. This suggests that the difference in urine 1-OHPG associated with these factors was not the same in smoke exposed and non-smoke exposed subjects. For example, among non-smoke exposed individuals, maté was associated with a significant increase in urine 1-OHPG. Among the smoke exposed there was no apparent increase in urine 1-OHPG. We obtained similar results with two different barbeque preparation variables, namely "Did you prepare barbeque in the last week" and "Do you prepare barbeque at least once a week."

**Figure 1:**
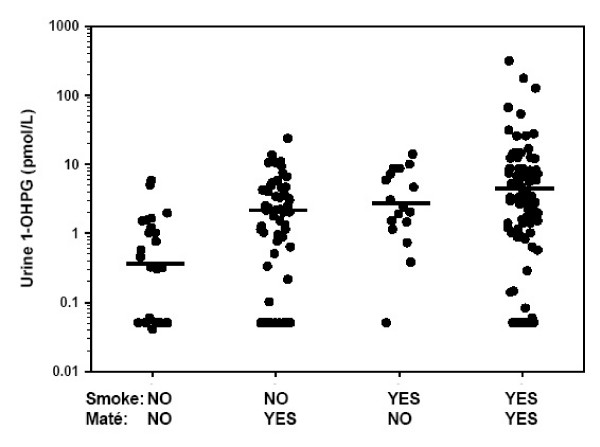
**Urine 1-hydroxypyrene glucuronide concentration plotted by maté consumption and tobacco smoke exposure**. We divided subjects into four groups by whether they ever consumed maté and whether they were smoke exposed (currently smoked tobacco or had a urine cotinine greater than category 1). Urine 1-OHPG concentration is plotted on the log scale. From left to right, the median for each group is indicated by the horizontal bar and the values are 0.3, 2.0, 2.4, and 3.4, respectively. From left to right, the number of subjects in each group is 28, 57, 17, and 97, respectively.

**Figure 2:**
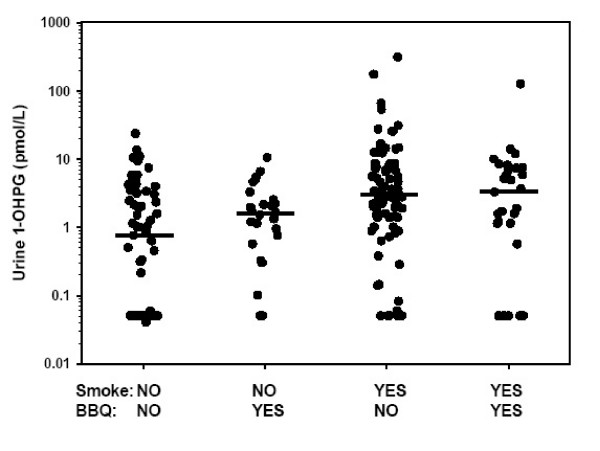
**Urine 1-hydroxypyrene glucuronide concentration plotted by frequency of barbeque preparation and tobacco smoke exposure**. We divided subjects into four groups by whether they prepared barbeque at least once a week and whether they were smoke exposed (currently smoked tobacco or had a urine cotinine greater than category 1). Urine 1-OHPG concentration is plotted on the log scale. From left to right, the median for each group is indicated by the horizontal bar and the values are 0.8, 1.7, 3.1, and 3.7, respectively. From left to right, the number of subjects in each group is 60, 25, 85, and 29, respectively.

Because of the complexity of the final model, we wished to examine the effect of maté consumption and barbeque preparation graphically. Therefore, we divided the cohort into groups and plotted the individual urine concentrations. Figure 1 presents the data by maté consumption and smoke exposure and Figure 2 presents the data by barbeque preparation and smoke exposure. In these figures, the effects of maté consumption and barbeque preparation are more pronounced in non-smokers.

We wanted to assure that our results were not due to the shape of the distribution of urine 1-OHPG concentrations, so we created an alternative urine 1-OHPG scale by categorizing subjects into five quintiles where the first quintile was subjects at or near the limit of detection and the remaining subjects were assigned to the remaining values. Replacing the log-transformed urine 1-OHPG concentration value with the quintile category and fitting the same final model produced very similar results, reassuring us that the results were not sensitive to the shape of the distribution (data not shown).

## Discussion

According to the Brazilian Ministry of Health, southern Brazil has incidence rates for ESCC of approximately 20.4/100,000/year for men and 6.5/100,000 for women. These rates are much higher than those observed in most western countries. Several epidemiologic studies have examined potential etiologic factors other than tobacco and alcohol in this region of South America that may contribute to the high rates of ESCC. Some evidence suggests that high consumption of *churrasco *and hot maté could be additional important risk factors [4-10].

The median urine 1-OHPG level of the inhabitants of southern Brazil who were examined in this study (2.09 pmol/ml) was similar to those found in two other high ESCC-risk areas, namely Linxian, China (2.06 pmol/ml) [16] and northeastern Iran (4.2 pmol/ml)[28]. All of these concentrations are much higher than those reported for non-smoking US residents (0.23 pmol/ml)[29]. As expected, tobacco smoking in our population had a significant association with urine 1-OHPG. Non-smoke exposed subjects who regularly prepare barbeque also had elevated urine 1-OHPG concentrations, presumably from increased smoke exposure during this activity. Surprisingly, we also found that any maté consumption significantly increased urine 1-OHPG concentrations and that there was a step-wise increase in 1-OHPG concentration with the volume of maté consumed.

Brazil is a country with recognized regional socio-economical and cultural differences. Rio Grande do Sul State has an economy based largely on agriculture and cattle production which has led to high consumption of red meat, due to relatively low prices and the availability of this product, and a preference for barbequed meat. The *churrasco *maker is exposed to coal or wood smoke when preparing the meat. We did not see a significant association between urine 1-OHPG concentration and the amount of barbeque consumed, but exposure to other potentially hazardous compounds that may be present in the barbequed meat, such as heterocyclic amines, should also be investigated [30].

Several epidemiologic studies in this region of South America have shown an association between maté consumption and risk of esophageal cancer [4-10]. Possible reasons for this association include ingestion of carcinogens present in the unprocessed leaves of *Ilex paraguayensis*, ingestion of carcinogens produced or added as contaminants during the processing of the leaves, and thermal injury to the esophageal mucosa caused by drinking maté tea at very hot temperatures. Many people in this region drink large amounts of maté at very high temperatures. In our study population, we saw a median intake (interquartile range) of 500 (100 – 1000) mls/day. Previous studies in southern Brazil have reported mean maté consumptions of 1200 and 1800 ml/day and mean temperatures measured just before consumption of 63.4 and 69.5°C [31,32]. Several studies have reported that only the temperature at which maté was drunk was significantly associated with ESCC risk, while the amount of maté consumed and the temperature at which it was extracted were inconsequential [7,11]. Other studies, however, reported that high temperature and a high volume of consumption were both important, and were independently associated with significantly increased risk of ESCC [4,8]. Yet another study reported that duration and amount of maté consumption was consistently associated with cancer risk, but temperature was not [5]. We did not collect information on mate temperature in this study because we had no *a priori *reason to suspect that consumption temperature would affect urine 1-OHPG concentration. We also thought that without objective temperature measurements, questionnaire data concerning mate temperature would not be sufficiently reliable for meaningful evaluations.

In most studies from this region, mate consumption is considered to be an independent risk factor for esophageal cancer. The underlying mechanism, whether thermal or chemical, remain unclear. Fonseca *et al*. reported that extracts of unprocessed *Ilex paraguayensis *are mutagenic in bacterial assays and can cause chromosomal aberrations in human peripheral lymphocytes treated *ex vivo *[33]. The processing of this herb for maté involves roasting the leaves over an open fire, which can lead to the formation or addition of PAHs or other contaminants. A single study of processed mate purchased in Germany reported that the leaves contained up to 461 μg/kg benzo[a]pyrene, but there were relatively low concentrations of this PAH in the prepared beverage [34]. Differences in maté brand and the details of tea preparation might change the amount of benzo[a]pyrene in the tea. The finding of benzo[a]pyrene in maté implies, however, that elevated 1-OHPG concentrations in maté drinkers may come directly from the maté, and need not be attributed to uncontrolled confounders such as smoking. Our findings raise the possibility that PAH exposure from consumption of maté may be one cause for the previously reported association between maté drinking and ESCC risk.

## Conclusion

We conclude that people in southern Brazil are exposed to high levels of PAH from tobacco smoke and maté drinking, as well as barbeque preparation and other unmeasured sources, and that this exposure may contribute to the high rates of ESCC observed in this area. Additional studies are needed to characterize this exposure more fully and to determine if it is etiologically associated with the high esophageal cancer rates found in this area.

## Competing interests

The author(s) declare that they have no competing interests.

## Authors' contributions

RBF designed the questionnaires and supervised the field studies. PTS supervised urine 1-OHPG measurements. CCA and FK contributed to the statistical analysis, MJR, PRT and SMD with the other authors contribute on the interpretation of the data and preparation of the manuscripts. All authors read and approved the final manuscript.

## Pre-publication history

The pre-publication history for this paper can be accessed here:



## Figures and Tables

**Table 1:** Characteristics and univariate comparisons of urine 1-hydroxypyrene glucuronide (1-OHPG) concentration in 199 healthy subjects from Rio Grande de Sul

**Characteristic**	**Category N (%)**		**1-OHPG (pmol/ml) median (IQR)**	***P*-value **[1]
Age quartile 1	<= 35	52 (26%)	1.90 (0.23–3.55)	0.027
Age quartile 2	>35	49 (25%)	1.90 (0.76–5.58)	
Age quartile 3	>45	52 (26%)	3.65 (1.36–7.58)	
Age quartile 4	>58	46 (23%)	1.55 (0.22–5.01)	
Sex, N (%)	Males	98 (49%)	2.19 (1.02–4.95)	0.52
	Females	101 (51%)	2.03 (0.06–7.23)	
Residence, N (%)	Rural	35 (18%)	1.97 (0.76–7.49)	0.91
	Urban	160 (82%)	2.16 (0.48–5.30)	
Ever drink maté, N (%)	No	45 (23%)	1.02 (0.05–2.03)	0.0004
	Yes	154 (77%)	2.70 (0.89–6.92)	
Maté quartile 1	<100 mls/day	53 (27%)	1.01 (0.05–2.16)	0.0001
Maté quartile 2	>100 mls/day	66 (33%)	1.97 (0.33–5.58)	
Maté quartile 3	>500 mls/day	38 (19%)	3.24 (1.40–8.15)	
Maté quartile 4	>1000 mls/day	42 (21%)	4.06 (1.21–8.50)	
Current tobacco smoker, N (%)	No	103 (52%)	1.14 (0.05–3.17)	<0.0001
	Yes	96 (48%)	3.76 (1.52–8.57)	
Ever regular tobacco smoker, N (%)	No	71 (36%)	1.14 (0.05–2.35)	<0.0001
	Yes	128 (64%)	3.41 (1.09–7.88)	
Urine cotinine, N (%)	0 (<1–10 ng/mL)	49 (25%)	0.89 (0.05–2.35)	<0.0001
	1 (10–30)	41 (21%)	1.52 (0.05–4.00)	
	2,3,4 (30–500)	39 (20%)	1.40 (0.29–3.68)	
	5 (500–2000)	36 (18%)	4.89 (1.74–8.69)	
	6 (>2000)	34 (17%)	7.36 (2.66–7.36)	
Smoke Exposure^2^	No	85 (43%)	1.14 (0.051–3.17)	<0.0001
	Yes	114 (57%)	3.30 (1.40–8.15)	
Ever prepare BBQ, N (%)	No	107 (54%)	2.03 (0.08–5.58)	0.31
	Yes	92 (46%)	2.136 (0.92–6.22)	
Prepared BBQ in the last week, N (%)	No	148 (74%)	2.09 (0.31–5.60)	0.63
	Yes	51 (26%)	2.09 (0.76–6.60)	
Prepare BBQ at least once a week, N (%)	No	145 (73%)	2.22 (0.33–5.84)	0.88
	Yes	54 (27%)	1.94 (0.95–5.90)	
Ever eat BBQ well done, N (%)	No	56 (28%)	2.09 (0.24–7.52)	0.82
	Yes	143 (72%)	2.09 (0.57–5.58)	
Ever drink beer, N (%)	No	126 (63%)	2.44 (0.30–6.92)	0.92
	Yes	73 (37%)	2.03 (1.02–4.63)	
Ever drink wine, N (%)	No	168 (84%)	2.22 (0.05–6.66)	0.62
	Yes	31 (16%)	1.97 (1.27–3.30)	
Ever drink cachaca, N (%)	No	165 (83%)	1.97 (0.33–5.39)	0.03
	Yes	34 (17%)	3.46 (1.52–8.19)	

[1] All *P*-values come from the Wilcoxon rank sum test and the two-sided normal approximation *P*, except cotinine and mate quartile p-values which came from the Kruskal-Wallis test.

[2] We defined smoke exposed subjects as subjects who are current tobacco smokers or have a cotinine value greater than category 1 (> 30 ng/mL).

**Table 2:** Multivariate associations^1 ^between characteristics and urine 1-hydroxypyrene glucuronide (1-OHPG) concentration in the healthy subjects from Rio Grande de Sul

**Characteristic**	**Estimate **[2]	**95% Confidence interval**	***P*-value **[3]
Intercept	-0.74	-1.56, 0.079	0.076
Male	-0.048	-0.31, 0.22	0.72
Urban residence	0.028	-0.28, 0.33	0.86
Ever drink cachaHa	0.20	-0.13, 0.54	0.23
Age quartile 1	Reference		
Age quartile 2	0.097	-0.23, 0.42	0.56
Age quartile 3	0.40	0.082, 0.72	0.014
Age quartile 4	0.17	-0.17, 0.50	0.33
Smoke Exposure^4^	0.98	0.045, 1.52	0.0004
Maté	0.53	0.16, 0.90	0.0053
Interaction (Smoke * Maté)	-0.51	-1.05, 0.10	0.11
Prepare barbeque weekly	0.21	-0.20, 0.62	0.32
Interaction (Smoke * BBQ)	-0.48	-1.026, 0.015	0.057

[1] All estimates come from a single multivariate linear regression model. The total model r^2 ^was 0.21

[2] The model was fit using log_10 _transformed urine 1-OHPG concentrations.

[3] All *P*-values come from F-tests.

[4] Subjects reporting current smoking or a cotinine value greater then 1 were considered smoke exposed.
